# Bilingual and monolingual children prefer native-accented speakers

**DOI:** 10.3389/fpsyg.2013.00953

**Published:** 2013-12-23

**Authors:** André L. Souza, Krista Byers-Heinlein, Diane Poulin-Dubois

**Affiliations:** Department of Psychology, Concordia UniversityMontréal, QC, Canada

**Keywords:** bilingualism, foreign-accented speech, social biases, children

## Abstract

Adults and young children prefer to affiliate with some individuals rather than others. Studies have shown that monolingual children show in-group biases for individuals who speak their native language without a foreign accent (Kinzler et al., 2007). Some studies have suggested that bilingual children are less influenced than monolinguals by language variety when attributing personality traits to different speakers (Anisfeld and Lambert, 1964), which could indicate that bilinguals have fewer in-group biases and perhaps greater social flexibility. However, no previous studies have compared monolingual and bilingual children's reactions to speakers with unfamiliar foreign accents. In the present study, we investigated the social preferences of 5-year-old English and French monolinguals and English-French bilinguals. Contrary to our predictions, both monolingual and bilingual preschoolers preferred to be friends with native-accented speakers over speakers who spoke their dominant language with an unfamiliar foreign accent. This result suggests that both monolingual and bilingual children have strong preferences for in-group members who use a familiar language variety, and that bilingualism does not lead to generalized social flexibility.

## Introduction

Language plays a fundamental role in how we divide the world into social categories. The linguistic features that speakers adopt such as their word choice, intonation pattern, speech rate, and accent influence the social attitudes of their listeners (Giles et al., 1987; Cargile et al., 1994). Language influences social evaluations and preferences early in life (Bigler et al., 1997; Kinzler et al., 2007, 2009, 2012). Most research to date has investigated monolingual children's social preferences. However, these children's development may not be representative of the many children who grow up in multilingual environments (Werker and Byers-Heinlein, 2008). Little is known about how early bilingualism influences children's social preferences. More specifically, there is virtually no research on how accented speech influences bilingual children's social preferences.

Accent—defined as the manner of pronunciation that is particular to an individual or group of individuals—is one of the most prominent linguistic cues used in forming social categories (Edwards, 1997). Like many other linguistic features, accent can influence the listener's judgments and perceptions about several traits of a speaker. Indeed, accent has been shown to influence adults' perceptions of competence, social status, intelligence, confidence, guilt, success, and fluency (Ryan and Giles, 1982). Studies have shown that adults evaluate non-accented speakers more favorably across these different traits compared to their accented counterparts (Giles and Sassoon, 1983; Seggie, 1983; Giles and Coupland, 1991; Carlson and McHenry, 2002; Dixon et al., 2002).

Recent developmental research has suggested that language and accent affect social preferences even early in life. In a series of experiments, Kinzler et al. (2007) found that 5–6 month-old infants preferred to look at speakers of their native language over speakers of a foreign language. In addition, they found that 10-month-old infants preferred to interact with a speaker who spoke their native language: English-learning infants reached for a toy offered by an English-speaking adult while French-learning infants reached for a toy offered by a French-speaking adult. Five-year-olds also displayed a preference for speakers of their native language over speakers of a foreign language. Children were shown pictures of two individuals, and then played a voice clip that spoke either their native language or a foreign language. When asked who they would prefer to be friends with, children most often chose the native language speaker.

Preferences for native language speakers have also been found in studies investigating children's social evaluations of accented speakers. While children can distinguish between a variety of accents, including regional, and foreign accents, foreign accents appear to be particularly salient (Girard et al., 2008). A study with 5-year-olds showed that children preferred to be friends with a native-accented speaker over a foreign accented speaker (Kinzler et al., 2007).

Not only do accents affect children's friendship preferences, but they also affect children's willingness to imitate and learn from different individuals. Kinzler et al. (2011) presented 4- and 5-year-old children with videos of two speakers: a native English speaker and a Spanish-accented English speaker. After hearing each individual speak for a few minutes, children were presented with a novel object whose function was unclear. Each speaker silently pantomimed a function for the novel object, and children were then asked which of the two functions they endorsed. Children endorsed the function demonstrated by the native-accented speaker more often than they endorsed the function demonstrated by the foreign-accented speaker. This effect was present even in a condition where the speakers produced nonsense speech with a native versus a foreign accent, suggesting that the effect was related to accent and not just to comprehensibility.

There is also evidence that children weigh accent more heavily than other cues to group membership such as race. When presented with a picture of a silent child, 5-year-old monolingual children preferred to be friends with same-race children. However, they chose other-race children when the same-race children spoke with a foreign accent (Kinzler et al., 2009). In other words, they preferred to be friends with a child from a different race than to be friends with a child that spoke with a foreign accent.

Together, these findings demonstrate that language and accent influence children's social preferences across a variety of tasks. However, these studies have all been conducted with monolingual children. Many children in the world grow up exposed to two or more languages, an experience that might even be more typical than exposure to a single language (Lieberson, 1981; Cohen and Haun, 2013). It is still not entirely clear how bilingualism interacts with social preferences. However, there are at least two reasons to expect bilinguals to show different social preferences than monolinguals. First, bilinguals have a more diverse linguistic experience, as they regularly interact with individuals who speak different languages. Because caregivers of bilingual children are likely to be bilingual themselves (De Houwer, 2007; Byers-Heinlein, 2013), it has been argued that bilingual children have more exposure to foreign-accented speech than monolinguals do (Bosch and Ramon-Casas, 2011; Byers-Heinlein and Fennell, 2013). Bilinguals might therefore be more accepting of the atypical pronunciations that characterize accented speech. Second, analogously to how regular switching between different languages enhances bilingual children's cognitive flexibility (Poulin-Dubois et al., 2011; Bialystok et al., 2012; but see also Paap and Greenberg, 2013 for counter-evidence from studies with adults), it is possible that regular switching between social partners speaking different varieties might generally enhance bilingual children's social flexibility.

Although no studies to date have examined how bilingualism affects children's social preferences for accented speakers, there is some evidence that exposure to multiple language varieties might affect other language-based in-group preferences. For instance, Kinzler et al. (2012) investigated how social preferences interact with exposure to a second language and a language's social status. They showed that 5–11 year-old monolingual South African children preferred speakers of their own language (Xhosa) over speakers of another language (French). However, for children attending school in English this pattern was reversed: these children preferred speakers of English (a high-status language) over speakers of Xhosa (their native language). This finding suggests that systematic exposure to another language can alter in-group biases. However, because language familiarity was confounded with social status, and participants' level of bilingualism was not directly measured, these results cannot speak specifically to the effects of bilingualism on social preferences.

Cohen and Haun (2013) examined the effects of exposure to multiple accents in monolingual children living in Brazil. Children were shown puppets, one who spoke in an accent that matched the child's accent, and one who spoke with a different regional accent, and were asked which puppet they would prefer to share a sweet with. An in-group bias (i.e., a preference for sharing with the native-accented puppet) was found only amongst the oldest children (9–10 years) and only for children living in a multi-accent environment. Although this study did not examine bilingualism *per se*, the results suggest that children's exposure to different speech varieties could influence their social preferences.

Finally, Anisfeld and Lambert (1964), using a match-guise paradigm, examined how monolingual and bilingual school-aged children attributed characteristics to speakers of different language varieties. They reported that monolinguals were more influenced by the speaker's language than bilinguals were. However, this study did not measure how bilingualism affected children's social preferences.

To begin to investigate how bilingualism affects children's social preferences, the present study compared how monolingual and bilingual children evaluate individuals with an unfamiliar foreign accent compared to those with a native accent. Participants were 5- and 6-year-old English and French monolinguals and English-French bilinguals. Their social preferences for native-accented versus foreign-accented (Haitian Creole) speakers of their dominant language were compared. We predicted that if bilingual children show generalized flexibility relative to monolingual children, then bilingual children should show an attenuated in-group bias relative to monolingual children. That is, bilinguals should show no preference for the native-accented over the foreign-accented speaker, while monolinguals should prefer the native-accented speaker, replicating previous results. However, if bilingual children's social preferences are driven by their experience with a particular accent (i.e., familiarity with the native language, and unfamiliarity with Haitian Creole), then both monolingual and bilingual children should prefer the native-accented speaker.

## Method

### Participants

Forty-four 5- and 6-year-old children (22 females, *M_age_* = 5 years and 8 months, range: 5 years and 1 month; 6 years and 8 months) participated in the study. Children were recruited from a local database of families interested in research, and from kindergarten classes at local private schools in Montréal, Canada. Children's language exposure and language proficiency was measured using a modified version of the LEAP-Q measure (Marian et al., 2007), which was completed by their parents. Parents answered questions about their children's exposure to and proficiency in each language. For example, parents were asked to indicate at what age their child's exposure to each language began, to estimate the percentage of time that their child was exposed to each language, and to rate their children's speaking and comprehension proficiency in each language. Because children were growing up in a bilingual city, almost all children had at least some exposure to a second language. Thus, following other research with similar populations (e.g., Bosch et al., 2013), children with 75% or more exposure to either English or French were classified as monolinguals (*N* = 24), and children with less than 75% exposure to their dominant language (English or French) were classified as bilinguals (*N* = 20). As such, most bilinguals had at least 25% exposure to English and 25% exposure to French, except for a few children who had a small amount exposure to a third language (< 20%). Children exposed to more than 20% of a language other than English or French (*N* = 14), or with any reported exposure to Haitian Creole (*N* = 1) were excluded from the analysis. Of the children included in the study, 20 (11 bilinguals) were English dominant and 24 (9 bilinguals) were French dominant.

The monolingual children had an average of 91.5% (*SD* = 10.0) exposure to their dominant language. The bilingual children had an average of 49.3% (*SD* = 14.4) exposure to English, 45.2% (*SD* = 16.4) exposure to French, and 5.9% (*SD* = 5.9) exposure to a third language. Nine were simultaneous bilinguals (exposed to both English and French since birth as first languages) and 11 were sequential bilinguals (exposed to their first language since birth, and their second language sometime after birth). The average age of acquisition of the second language was 2.63 years (range: 1–5).

Speaking and comprehension proficiency were assessed via parental report on a 0–10 scale where 0 was no proficiency and 10 was perfect proficiency. Monolingual children had an average speaking proficiency of 8.9 (*SD* = 2.20) in their dominant language, and an average comprehension proficiency of 9 (*SD* = 2.21) in their dominant language. For speaking, bilingual children had an average proficiency of 9 (*SD* = 1.94) in English, and 7.79 (*SD* = 2.39) in French. For comprehension, the bilinguals had an average proficiency of 9.15 (*SD* = 1.49) in English and 8.57 (*SD* = 1.95) in French.

### Materials

#### Auditory stimuli

Eight native speakers of English, eight native speakers of French and eight native speakers of Haitian Creole (half male and half female) recorded eight declarative sentences, for example “*The sun shines in the sky*.” The native speakers of English recorded the sentences in English, the native speakers of French recorded the sentences in French, and the Haitian Creole speakers recorded the sentences in both English and French. Haitian Creole was chosen as the foreign accent because many Haitians in Montréal are trilingual in Creole, English, and French, and thus the same foreign accent could be used in all conditions.

To ensure that the sentences were indeed perceived as native-accented and foreign-accented, 20 undergraduate students (French or English dominant) rated the level of accentedness for all the sentences in their dominant language. That is, English-dominant students rated the English sentences whereas French-dominant students rated the French sentences. They used a Likert scale ranging from 1 (not accented) to 7 (strongly accented). The results showed that sentences produced by native speakers were perceived as significantly less accented (*M* = 2.33, *SD* = 1.12) than those produced by the non-native speakers (*M* = 5.26, *SD* = 1.23), *t* (19) = −6.93, *p* < 0.001 (Cohen's *d* = 2.48).

#### Visual stimuli

Photographs of 16 smiling adults (half male, half female) of European heritage were used as visual stimuli. As none of the children had previous experience with Haitian Creole speakers, they were unfamiliar with their typical ethnic characteristics. Thus, speaker ethnicity in the visual stimuli could be kept constant and dissociated from the true ethnicity of the speakers who recorded the auditory stimuli. Images were selected from the NimStim set of facial expressions (Tottenham et al., 2009), or were photos taken in a similar style. Images were paired together such that each pair was of the same gender, and was as similar as possible in other features (e.g., hair color and style).

### Procedure

The procedure was modeled after Kinzler et al. (2007). Participants were tested individually in a quiet room either in the laboratory or at their school. All participants were tested in their dominant language by a female bilingual experimenter, who had grown up hearing and speaking both languages and had a native accent in both languages. All experimental trials were presented in the participant's dominant language.

The experimenter told children that they were going to see pictures of people and hear their voices, and that they would need to pick the person that they would most like to be friends with. On each trial, the experimenter displayed a pair of faces on a laptop screen. The experimenter then pointed at once face and drew attention to it verbally, e.g. “Let's listen to him.” She then played an audio clip while the face loomed on the screen. This procedure was then repeated for the other face. Each pair of speakers uttered identical sentences, however one speaker had a native accent while the other had a foreign accent. A different sentence was used on each trial. The side of presentation of the native versus accented speaker and the order in which the two speakers were introduced was counterbalanced across trials for each child as well as across children, and the particular pairing of the face and the voice (native vs. foreign accent) was counterbalanced across children. Children were then asked to point to the person that they would most like to be friends with. Each child completed 8 trials. The number of times (out of 8 trials) that children chose the native-accented speaker was recorded and the proportion of native-accented speaker choices was used as the main dependent variable in the subsequent statistical analyses.

## Results

**Figure 1:**
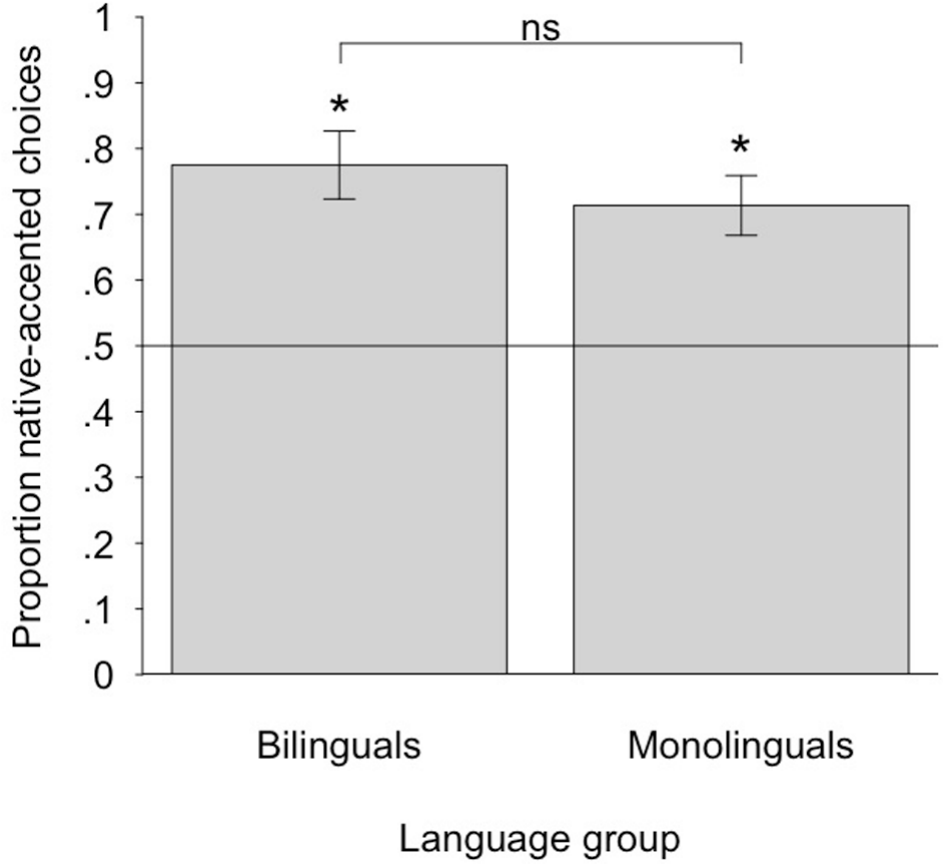
**Children's preference for native-accented speakers as a function of language group.** Error bars show the standard error of the mean. ^*^p < 0.001.

**Figure 2:**
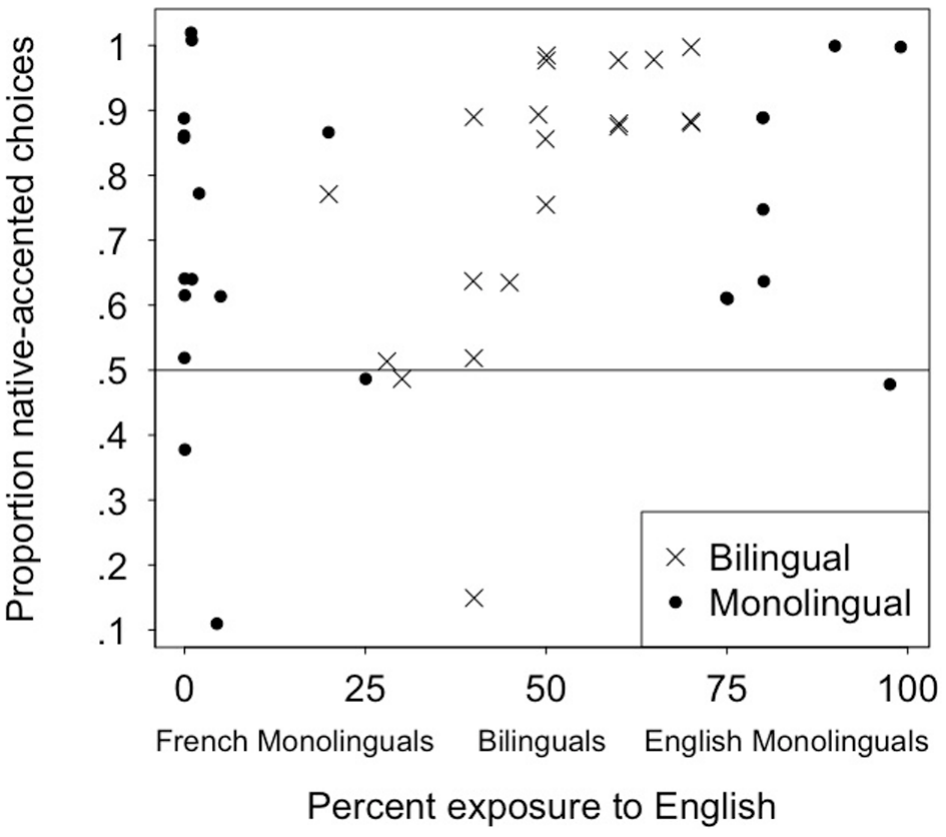
**Children's preference for the native-accented speaker as a function of language exposure**.

To test whether the speakers' accent influenced monolingual and bilingual children's social preferences, we conducted two-tailed one-sample *t*-tests comparing the proportion of native-accented speaker choices against chance (0.5). Results showed that both the monolingual children (*M* = 0.71, *SD* = 0.22) and the bilingual children (*M* = 0.77, *SD* = 0.23) chose the native-accented speaker significantly more than chance: *t* (23) = 4.70, *p* < 0.001, Cohen's *d* = 0.95 for the monolinguals, and *t* (19) = 5.31, *p* < 0.001, Cohen's *d* = 1.18 for the bilinguals (means are displayed in Figure 1). Monolinguals and bilinguals did not differ significantly from each other, *t* (42) = 0.89, *p* = 0.38. A scatterplot of individual results is presented in Figure 2, showing that very few children preferred the foreign-accented speaker over the native-accented speaker. As is apparent in the figure, there was no evidence that children's preferences varied as a function of their language dominance, or the language of testing.

## Discussion

The present study compared 5–6 year-old English and French monolinguals' and English-French bilinguals' preferences for native-accented versus foreign-accented speakers of their dominant language. We presented monolingual and bilingual children a series of trials in which they heard two speakers, one who spoke their dominant language with a native accent and another who spoke it with an unfamiliar Haitian Creole accent, and asked them which person they wanted to be friends with.

Consistent with previous findings (Kinzler et al., 2007), monolingual children preferred the native-accented speaker, but contrary to our initial predictions, bilingual children showed the same preference for the native-accented speaker. This result replicates and extends previous research that reported that older monolingual children exposed to a range of languages and accents are also biased toward native-accent speakers, particularly at older ages [9–10; Cohen and Haun (2013), see also Lev-Ari and Keysar (2010); for related work showing adults' preference for native accents]. Our findings suggest that bilingual children's greater exposure to different languages and accents does not necessarily lead to generalized social flexibility. In the current study, bilinguals were not more tolerant of foreign-accented speech than monolinguals. As we only tested 5-year-old children, future studies should test younger and older children using the same paradigm, to examine whether differences between monolinguals and bilinguals would be apparent at other ages.

Given the differences between our predictions and our results, the question remains as to what drives children's language-based preferences. One possibility is that, at least at younger ages, familiarity is a strong driver of preferences. That is, children prefer native speakers over foreign and foreign-accented speakers because the native-accented speech is more familiar. If this is the case, then bilingual children might show attenuated preferences to certain languages and accents because they have been exposed to these more than monolinguals. If so, English-French bilinguals might react similarly to a native speaker of French and an English-accented speaker of French. This comparison should be tested in future work. Consistent with our findings, this explanation predicts that when a language variety is unfamiliar to both monolinguals and bilinguals, both groups will prefer the unaccented speaker.

A second explanation is related to children's emerging ability to make social evaluations. A large literature on selective trust suggests that children make judgments of who to learn from and who to interact with (Mills, 2012). For example, children prefer to learn from those who have been accurate in the past (Harris and Corriveau, 2011), and 5-year-olds (but not younger children) value past accuracy over familiarity (Corriveau and Harris, 2009). Children may interpret a foreign accent as a cue that the speaker has incomplete or unreliable knowledge. However, a second heuristic proposed to underlie children's selective trust is group membership (Harris and Corriveau, 2011). Children prefer to learn from those who are part of their linguistic in-group (Kinzler et al., 2011). Both monolinguals and bilinguals in the current study might have detected the accented speaker as being an out-group member, and thus displayed a social preference for their in-group member.

The current data cannot tease apart these possibilities. However, future research with bilingual children could provide an important test of these two theoretical positions. For children exposed to a single language, membership in a linguistic group and familiarity with a language are confounded: monolingual children are typically only familiar with the language of their in-group. However, because bilingual children know multiple languages, these two can be dissociated. For example, a study of Mexican-American children showed that they varied in their allegiance to their Mexican heritage, despite being fully bilingual in English and Spanish (Schecter and Bayley, 1997). Future studies can test children who are equally familiar with and fluent in two languages, but who only affiliate with one of these as their in-group.

In sum, our results indicate that monolingual and bilingual 5–6 year-olds prefer to be friends with those who speak their language with a familiar native accent than with those who speak with an unfamiliar foreign accent. Thus, simple exposure to multiple language varieties does not confer bilingual children with generalized social flexibility. However, this finding does not rule out the possibility that bilingualism might lead to a more circumscribed flexibility with respect to the varieties that are familiar to them. Future research with children of different ages, language proficiencies, and group membership identities that compares preferences across a number of language varieties will be instrumental to further clarifying the origins of language-based social preferences.

### Conflict of interest statement

The authors declare that the research was conducted in the absence of any commercial or financial relationships that could be construed as a potential conflict of interest.
